# Degradation effects of reused PA12 powder in selective laser sintering on material characteristics, dimensional accuracy and mechanical strength

**DOI:** 10.1038/s41598-025-20280-7

**Published:** 2025-09-30

**Authors:** Valentina Vendittoli, Maria Cristina Mascolo, Wilma Polini, Michael S. J. Walter, Luca Sorrentino, Alexandru Sover

**Affiliations:** 1 Department of Civil and Mechanical Engineering, University of Cassino and Southern Lazio, Via G. Di Biasio 43, 03043 Cassino, Italy; 2 Department of Literature and Philosophy, Via S. Angelo – Località Folcara, 03043 Cassino, FR Italy; 3 Faculty of Engineering, University of Applied Sciences Ansbach, Residenzstr. 8, 91522 Ansbach, Germany

**Keywords:** Additive manufacturing, Selective laser sintering, PA12, Degradation, Powder reuse, Mechanical engineering, Mechanical properties

## Abstract

Selective Laser Sintering of Polymers is a widely used Additive Manufacturing technology that involves a laser to selectively sinter layers of a powder bed, with Polyamide 12 being a common material choice. Despite its favourable processability and component performance, the printing process leaves a significant amount of unsintered powder that undergoes heat treatment due to temperature gradients during printing, leading to material degradation over time. A deep comprehension of the aging behaviour in the powder for rightly planning the successive building process is thus necessary to define the proper recycling methods. This paper presents a comprehensive study of the thermal and structural characteristics of Polyamide 12 after five successive reusing cycles, as well as the dimensional accuracy and the mechanical strength of the corresponding printed parts. The study includes tests on the powder that underwent successive printing, and the parts manufactured using this powder. The results were compared to those obtained from virgin powder. These results were used to justify the differences in mechanical, macro-geometrical, and micro-geometrical performance between virgin and multiple reused powder parts. The results indicate that the powder degradation causes a significant reduction of the mechanical strength, and the texture quality of parts made from reused powder, while the dimensional accuracy remains very high.

## Introduction

The advancement of Additive Manufacturing (AM) has enabled the production of complex parts with varied features. However, Selective Laser Sintering (SLS), a widely used AM technique classified under the Powder Bed Fusion (PBF) family^1^, presents challenges related to material waste and powder degradation. In SLS, a polymer powder, preferred for its good dimensional and mechanical properties while requiring low laser power and processing temperatures, is selectively sintered by a laser to form a solid structure. Polyamide 12 (PA12) is the most often used semicrystalline polymer^2,3^.

A significant limitation of SLS is that nearly 80% of the powder remains unsintered after each print, undergoing multiple thermal cycles that contribute to material degradation. This degradation alters the chemical composition, crystallinity, and flowability, affecting printed parts’ mechanical performance and surface finish^4,5^. To mitigate these effects, various powder recycling strategies have been explored.

## State of the art

The most common recycling method is the powder refreshing with virgin powder at different percentages. Loper et al. studied different ratios in the mixture and successfully implemented an algorithm where only 30% of virgin powder is enough to enchant the mechanical properties in the printed specimens^6^. The melt flow rate test is used to verify if the mix is still printable. In this case, different range values can be applied to predict the possible surface quality of the parts^7^. This method does not consider the mechanical performance connected with the degradation described by the flow index. However, this solution will not be sustainable for an extended period. The mixture will contain powder with different reusing stages and characteristics, making it even more challenging to control recycling. Furthermore, the mixing method must be defined to ensure good powder homogeneity^8^.

Another approach to mitigating degradation involves the chemical modification of PA12 powder by adding chain splitters. Introducing 5 wt% chain splitters increases flowability, achieving results comparable to a 1:1 mixture of virgin and used powder. This improves surface quality, although some roughness persists due to residual chain splitter particles. While this method enhances mechanical performance, the tensile strength and elongation remain lower than parts produced using only virgin powder, yet still superior to those made from 100% used powder^9,10^.

A low-temperature laser sintering (LTLS) technique has been developed to minimise powder degradation by reducing the powder bed temperature to 140 °C. This process subjects the unsintered powder to less thermal stress, preserving its properties over multiple cycles^11^. However, to compensate for the lower sintering temperature, the laser energy must be increased, and the printing strategy must be adapted to suit the geometry of the printed parts, ensuring effective sintering. Despite these improvements, LTLS introduces new challenges. Parts produced using this method require rigid fixation to prevent curling, a phenomenon caused by the lower processing temperatures^12^.

The high thermal gradient between the melt pool and the powder bed surface can also lead to premature recrystallisation, negatively impacting process stability and mechanical performance^13^. While various methods have been developed to counteract powder degradation, none completely restore the material’s original properties. In the case of LTLS, additional factors can further influence part performance, sometimes leading to undesirable effects. Understanding the ageing process of PA12 powder at different reuse stages is critical for developing sustainable recycling strategies. The degradation of powder properties over multiple reuse cycles directly affects mechanical performance, dimensional accuracy, and surface quality, ultimately influencing the reliability of Selective Laser Sintering (SLS) as an industrial manufacturing process. Despite its widespread adoption, a significant gap remains in understanding how powder degradation correlates with final part properties. To address this, eight consecutive printing cycles were conducted using PA 2200 powder with a Formiga P110 printer. The powder was examined at the virgin, second, fourth, sixth, and eighth reuse cycles to assess structural and chemical changes. With each reuse, particle cracking increased, and prolonged exposure to high temperatures led to the evaporation of water and alcohol, altering powder composition. Additionally, the number of fine particles increased due to fragmentation, reducing powder flowability and negatively impacting the surface finish of printed parts^14,15^. To further explore these effects, the same batch of PA12 powder was subjected to annealing treatments under varied conditions in a nitrogen environment to simulate different thermal exposures. Results showed that crystallinity progressively decreased after the third reuse cycle, whereas molecular weight exhibited an opposite trend, creating additional challenges for powder recycling and process optimisation^16,17^.

Most prior investigations into PA12 sintering and powder degradation have focused on high-power CO_2_ laser-based SLS systems, such as those developed by EOS, which operate at wavelengths near 10.6 µm and are well-suited for processing polyamide powders due to their strong infrared absorption characteristics^9^. These systems are known to generate deep melt pools and uniform sintering behaviour, resulting in high mechanical performance and low porosity parts^18^. The printers typically operate in nitrogen-inert atmospheres and benefit from highly optimized thermal control and process repeatability^16,19,20^.

By contrast, other SLS systems, including increasingly used compact and research-oriented platforms, employ diode lasers operating in the near-infrared range (e.g., 808–980 nm). These exhibit different energy-material interactions due to lower intrinsic absorption by standard PA12 powders^21^. Variations in beam penetration depth, energy density, and thermal profiles across these systems can influence powder fusion, degradation dynamics, and powder reuse behaviour^22^.

While the present study was conducted using a diode-laser-based platform, the insights into powder ageing, material properties, and reuse trends remain broadly relevant for understanding sintering behaviour under varying thermal and optical conditions. The specific laser type and process window should nonetheless be considered when comparing results across different SLS configurations. The diversity in SLS hardware reinforces the importance of linking material behaviour not only to powder characteristics but also to the energy delivery and absorption profile inherent to the chosen laser system^23,24^.

In addition to laser type, the processing atmosphere plays a key role in powder degradation. Most industrial SLS systems operate in nitrogen-purged chambers that reduce oxidative degradation, especially at high processing temperatures. In contrast, the Sinterit Lisa system used in this study operates in ambient air without gas shielding, subjecting the powder to oxidative exposure during every cycle. This open-air condition accelerates ageing phenomena such as cross-linking, increased viscosity, and porosity formation even after a few reuse cycles^4,17^.

This distinction is crucial, as it means powder degradation mechanisms in low-cost, open systems differ significantly from those in nitrogen-controlled setups. The outcomes of this study are therefore particularly relevant for understanding and improving material reuse strategies in compact, accessible SLS systems that are increasingly used in research and small-scale production contexts.

However, there is a lack of systematic studies on the effect of the degradation on the material characteristics of powder and printed parts, the mechanical performances of printed parts as well as the quantification of dimensional accuracy and surface quality in terms of roughness under consideration of the successive reuse of powder.

This paper presents an in-depth experimental analysis of the extensive reuse of the PA12 powder and the transfer of degradation effects from the powder to the part during the printing process in an oxygenated environment. It investigates the effects of the degradation on the material characteristics, the dimensional accuracy and the roughness as well as on the mechanical strength of the printed parts. A statistical analysis of the impact of the degradation on the dimensional accuracy, roughness and mechanical strength of the printed parts was implemented. These insights will contribute to developing optimised recycling strategies, ensuring that reused powder can achieve properties comparable to virgin powder, thus promoting a more sustainable and cost-effective additive manufacturing process.

This paper is organised as follows: “Methodology” section outlines the methodology step-by-step and provides further details and relevant parameters and settings of each step. “Results” section presents the experimental results, including findings on powder degradation, dimensional accuracy, surface roughness, and the corresponding statistical analysis of the results. Additionally, the section discusses the outcomes of mechanical testing and their implications for part performance. “Discussion” section summarises the conclusions and future research directions, providing insights into how SLS powder recycling can be optimised for a more sustainable manufacturing process.

## Methodology

**Fig. 1:**
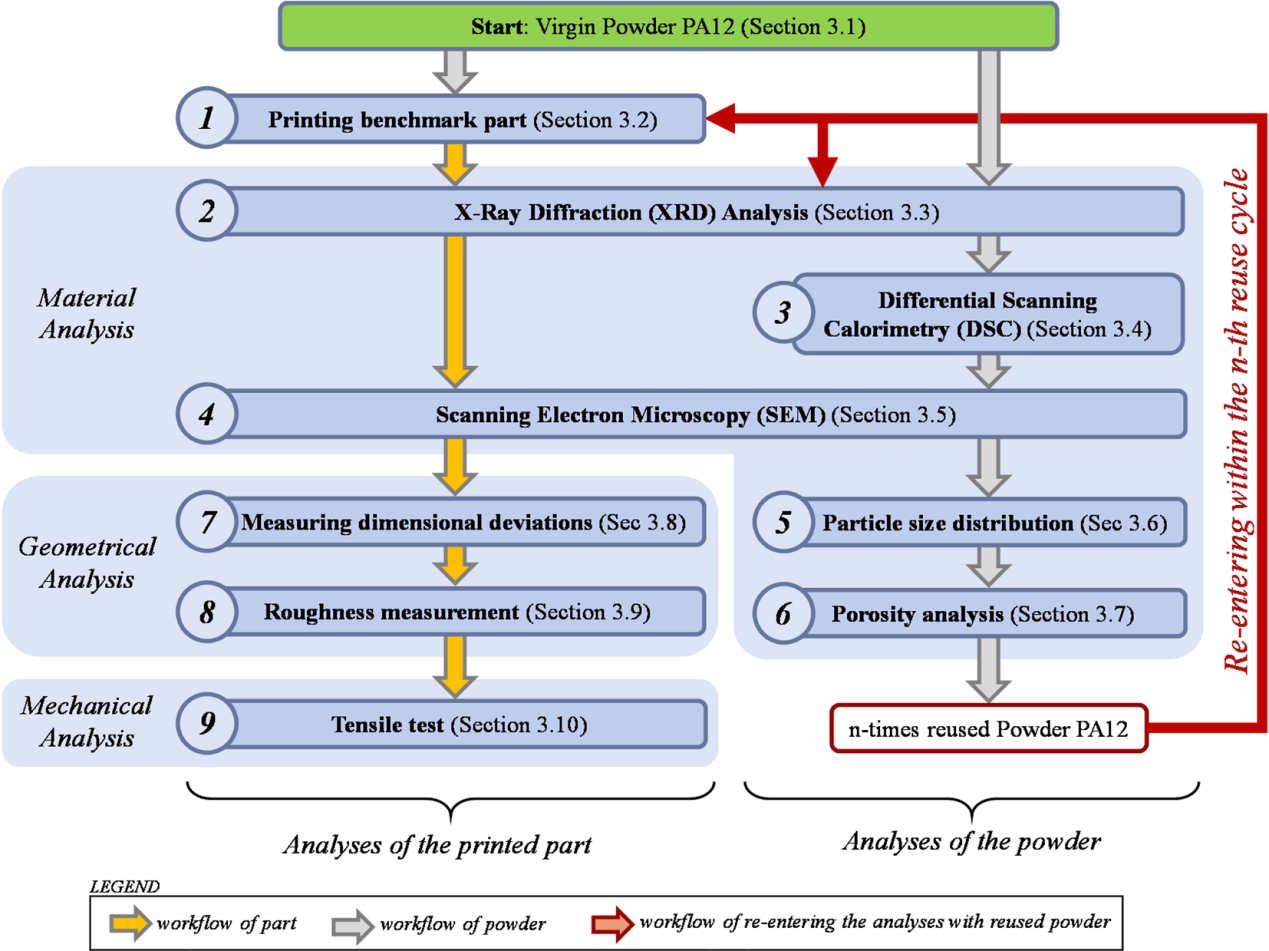
Methodology.

The methodology adopted for this study involves a detailed analysis of the PA12 powder as well as of the resulting parts from SLS-printing with the powder. A range of experimental techniques are applied to investigate the effect of powder degradation on the material characteristics, the dimensional accuracy and the mechanical strength. All nine individual steps are put in order and classified in Fig. 1 and summarise in the following list:

- **Step 1**: Benchmark Part Production: Virgin Sinterit PA12 powder was used to fabricate tensile specimens (ASTM D638-22 standard) using a Sinterit Lisa SLS printer under constant processing parameters to establish a baseline for comparison.
- **Step 2**: X-Ray Diffraction (XRD): The crystalline structure of both virgin and reused powder, as well as printed parts, was examined using a Philips XRD system. Diffraction patterns were used to assess phase changes over reuse cycles.
- **Step 3**: Differential Scanning Calorimetry (DSC): Thermal properties of the powder were analyzed using a Mettler Toledo DSC 3 + , with crystallinity calculated from heat flow data across six powder reuse stages.
- **Step 4**: Scanning Electron Microscopy (SEM): Morphological changes in the powder and parts were assessed using a Philips SEM system (XL series), highlighting particle damage and surface features.
- **Step 5**: Particle Size Distribution: Laser diffraction measurements (Malvern Mastersizer 2000) were performed according to ISO 13,320:2020 to assess shifts in powder size distribution due to thermal degradation.
- **Step 6**: Porosity Analysis: Bulk and solid densities were measured using a Fluometer ADP (ISO 60) and helium pycnometer (DIN 66,137–2), allowing porosity to be quantified as a function of reuse cycle.
- **Step 7**: Dimensional Accuracy: Printed part dimensions were measured with high precision using the KEYENCE IM-7000 optical system, with internal and external features assessed for deviation from the nominal CAD model.
- **Step 8**: Surface Roughness: Surface texture was evaluated on the top (Z-direction) surface using a Waveline W20 profilometer, following ISO 16,610–21, with a 2 μm tip and 0.8 mm cutoff.
- **Step 9**: Tensile Testing: Mechanical performance was tested using an MTS Criterion 43 system (5 kN load cell), following ASTM D638-22, with results reported in terms of ultimate tensile strength and elongation.

The experimental approach provides a comprehensive view of PA12’s behaviour (as part and powder) in its virgin form and after a cycle of reuse. It provides crucial data to optimise printing parameters and improve material strength in industrial applications considering powder degradation due to its multiple reuse.

### Starting point: characterization of the virgin powder PA12

The experiments were conducted using PA12 powder by manufacturer Sinterit (Poland), a commercial PA12 material widely used in SLS. The initial powder batch, henceforth called “virgin”, was characterised in its virgin state and subsequently analysed after multiple reuse cycles to investigate degradation effects. The main properties of the virgin PA12 powder are listed in Table 1.

**Table 1:** Properties of the virgin Sinterit PA12 powder ^25^.

Property and unit	Value
Particle size (μm)	18–90
Density of solid parts (g/cm^3^)	0.92
Melting temperature (°C)	185
Tensile strength (MPa)	32
Young’s modulus (MPa)	1470
Elongation at break (%)	10

While Sinterit PA12 Smooth is designed for compact SLS systems and features a broad particle size distribution (18–90 µm), EOS PA2200 has a narrower range (typically 45–60 µm), higher sphericity, and superior flowability. These differences impact sintering behaviour and powder reuse performance, especially under oxidative conditions. Therefore, the findings of this study apply primarily to materials formulated for low-power, open-atmosphere setups and should not be directly extended to powders optimized for nitrogen-shielded CO_2_-laser systems.

### Step 1: printing the benchmark parts

**Fig. 2:**
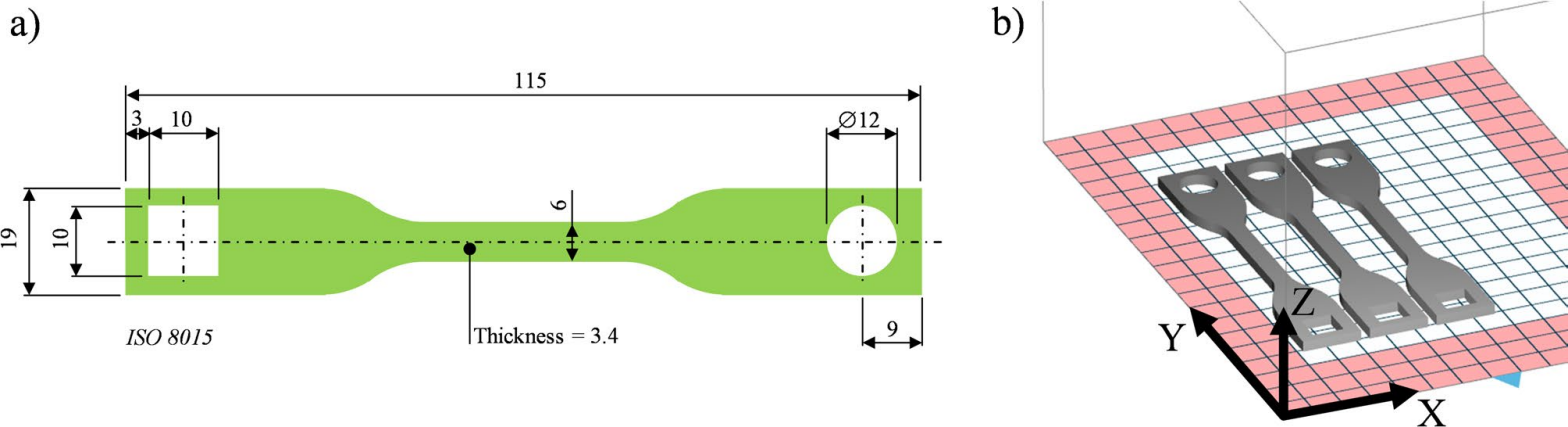
(**a**) Tensile specimen according to ASTM-D 638–22, all dimensions in mm^26^; (**b**) Pre-Processing: Preview of the part position and orientation in the print chamber.

To investigate the dimensions and mechanical properties of the printed parts, a tensile specimen was designed according to the ASTM-D 638–22 standard (see Fig. 2a). Two internal geometric features (one round and one square hole) were added to the model to provide additional measurement points for dimensional deviations of hollow structures. These modifications were applied specifically to the clamp-sections of the specimens to prevent any interference with the tensile test process.

The process was carried out using a Sinterit LISA SLS printer. Compared to high-end CO_2_ laser SLS systems such as EOS Formiga P110, which typically operate with 30–60 W CO_2_ lasers in nitrogen-purged chambers, the Sinterit Lisa uses a 5 W diode laser in ambient air. These conditions lead to significant differences in energy absorption, thermal gradients, and oxidation exposure, which affect both powder aging and part quality. Therefore, the results presented here are specific to the capabilities and constraints of compact diode-laser-based SLS platforms. The specifications of the printer are reported in Table 2^27^. The Sinterit LISA SLS printer was used to fabricate three specimens under controlled processing conditions. The processing parameters were kept constant throughout all print cycles to isolate the effects of powder degradation. All specimens were oriented in the same build direction to minimise variability caused by orientation effects.

**Table 2:** Technical specification of SLS-printer (model: Sinterit LISA).

Parameter and unit	Value
Build volume (mm*mm*mm)	150*200*150
Laser power (W)	5
Print bed temperature (°C)	5 below the melting temperature T_m_
Feed bed temperature (°C)	0–150
Print chamber temperature (°C)	0–140
Laser speed (mm/h)	up to 3
Layer height (mm)	0.075–0.175

The recommended printing parameters from the manufacturer were set for this project: the chamber temperature is set to 140 °C and the surface printing temperature to 178 °C. The layer height was 0.1 mm. All specimens were printed flat on the build plate (XY-surface in Fig. 2b). Six consecutive printing cycles were made with constant process parameters, starting from the virgin powder. Before every reuse, the powder collected from the printer was put in a sieve to remove any clumps^28^.

### Step 2: X-ray diffraction (XRD) analysis of part and powder

To quantify the effects of powder reuse on the material characteristics of parts and powder, five analytical techniques are applied to the powder after each reuse. The analysis was an X-Ray Diffraction (XRD) to evaluate the crystalline structure of the powder and part (Step 2 in Fig. 1). During an XRD analysis, a monochromatic X-ray beam is directed at a sample while varying the incidence angle. As the beam interacts with the atomic structure of the material, it undergoes diffraction according to Bragg’s Law^29^:

1 $$n\lambda = 2d\,sin\,\theta$$

where n is an integer, λ is the X-ray wavelength, d is the interplanar spacing, and θ is the diffraction angle (between the incident ray and the scatter plane). The virgin powder and the 5-times reused powder, as well as the corresponding printed parts, were analysed by a Philips XRD device. Each analysis run was repeated two additional times to ensure the reliability of the results. The diffracted beams were recorded as a function of 2θ, generating a diffraction pattern characteristic of the sample’s crystalline structure. The resulting diagrams are called XRD patterns and show peaks in the intensity (in counts) corresponding to constructive interference from specific atomic planes, allowing phase identification and structural analysis. These XRD patterns are shown in “Results of the material analyses of powder and parts (Steps 2–6)” section.

### Step 3: thermal analysis of the powder using differential scanning calorimetry (DSC)

The investigation of the thermal characteristics of the powder was done using the thermoanalytical technique of Differential scanning calorimetry (short: DSC). The principle of DSC is based on the measurement of the temperature-dependent difference in the amount of heat that is required to increase the temperature of a sample and reference as a function of temperature. During the entire experiment, the temperature of the sample and the reference are kept constant.

A Mettler Toledo DSC 3 + was used. Three powder samples for each of the six use cycles (virgin plus five times of reuse) were analysed, making a total of 18 experiments. Each sample weighs 10 ± 0.5 μg and is sealed in aluminium pans^30^. It was subjected to a heating ramp from 120 to 200 °C at a constant rate of 10 K/min. Then the temperature was kept constant for five minutes, then a cooling ramp with a constant rate of 10 K/min from 200 to 23 °C was applied. Finally, the temperature was kept constant for an additional five minutes. The measurements were carried out under a 30 mL/min nitrogen flow rate. To quantify the degradation effect of the printing process, the degree of crystallinity was calculated as follows:

2 $$Xc\left( \% \right) = \frac{{\Delta H_{f} }}{{\Delta H_{f}^{0} }} \cdot 100\%$$

where *ΔH*_*f*_ is the heat of fusion from the DSC results, and *ΔH*^*o*^_*f*_ is the heat of fusion of 100% crystalline PA12. The *ΔH*_*f*_ is 209.3 J/g, according to the literature^31^. The results of the 18 DSC analyses are individual values of relevant process parameters, such as the temperature of the crystallisation shoulder and the crystallisation temperature peak T_c_-peak. These are presented in “Results of the material analyses of powder and parts (Steps 2–6)” section.

### Step 4: scanning electron microscopy (SEM) of part and powder

The morphological analysis of the sample surface was performed by SEM, adopting a Philips microscopy (XL series, Almelo, Nederland). SEM is a widely recognised and extensively utilised imaging method that exploits the electrons emitted from a material’s surface when it is hit by a scanning electron beam. During the process, a finely focused electron beam is methodically moved across the target area. A crucial factor for contrast is how the backscattering coefficient varies with the average atomic number. As a result, areas containing elements with higher atomic numbers tend to appear brighter than those with lower atomic numbers, allowing for their visual differentiation in these images based on their atomic composition. Secondary electron (SE) mode was utilised to capture pictures of the surface due to the sample’s sensitivity to electrons, and to prevent charging, the samples were coated with a 20 nm layer of gold. The resulting images are called SEM micrographs and are shown in “Results of the material analyses of powder and parts (Steps 2–6)” section. ^32^

### Step 5: analysis of the particle size distribution of the powder

A Mastersizer 2000 from Malvern that uses laser diffraction^33^ was used to measure the powder’s size distribution according to the standard ISO 13,320:2020^34^. Five measurements were carried out for each powder batch, and the average was considered since the observed variation was not significant. The resulting volume distribution as well as its corresponding mean value are reported in “Results of the material analyses of powder and parts (Steps 2–6)” section.

### Step 6: analysis of the Porosity of the Powder

The bulk density tester Fluometer type ADP (manufacturer: Karg^35^) was used to calculate the bulk density ${\rho }_{bulk}$ of the powder, following the standard ISO 60^36^ as:

3 $$\rho_{bulk} = \frac{{w_{powder} }}{{V_{cylinder} }}$$

where w_powder_ is the weight of the powder in the cylinder, and V_cylinder_ is the volume of the cylinder, which is 100 cm^3^. Furthermore, the Helium pycnometer (model: pyconmatic ATC; manufacturer POROTEC^37^) was used to measure the solid density of the powder ${\rho }_{solid}$ according to the DIN 66,137–2:2019-03^38^. Therefore, the difference between the specific and bulk volume of a powder sample is measured. This method is based on the displacement principle, in which the powder is used as a solid and helium as the displaced medium^39^. The resulting porosity $\varepsilon$ of the powder is determined by:

4 $$\varepsilon = 1 - \frac{{\rho_{bulk} }}{{\rho_{solid} }}*100$$

where ${\rho }_{bulk}$ is the density of the bulk and ${\rho }_{solid}$ is the solid density.

### Step 7: analysis of the Dimensions of the Part

**Fig. 3:**
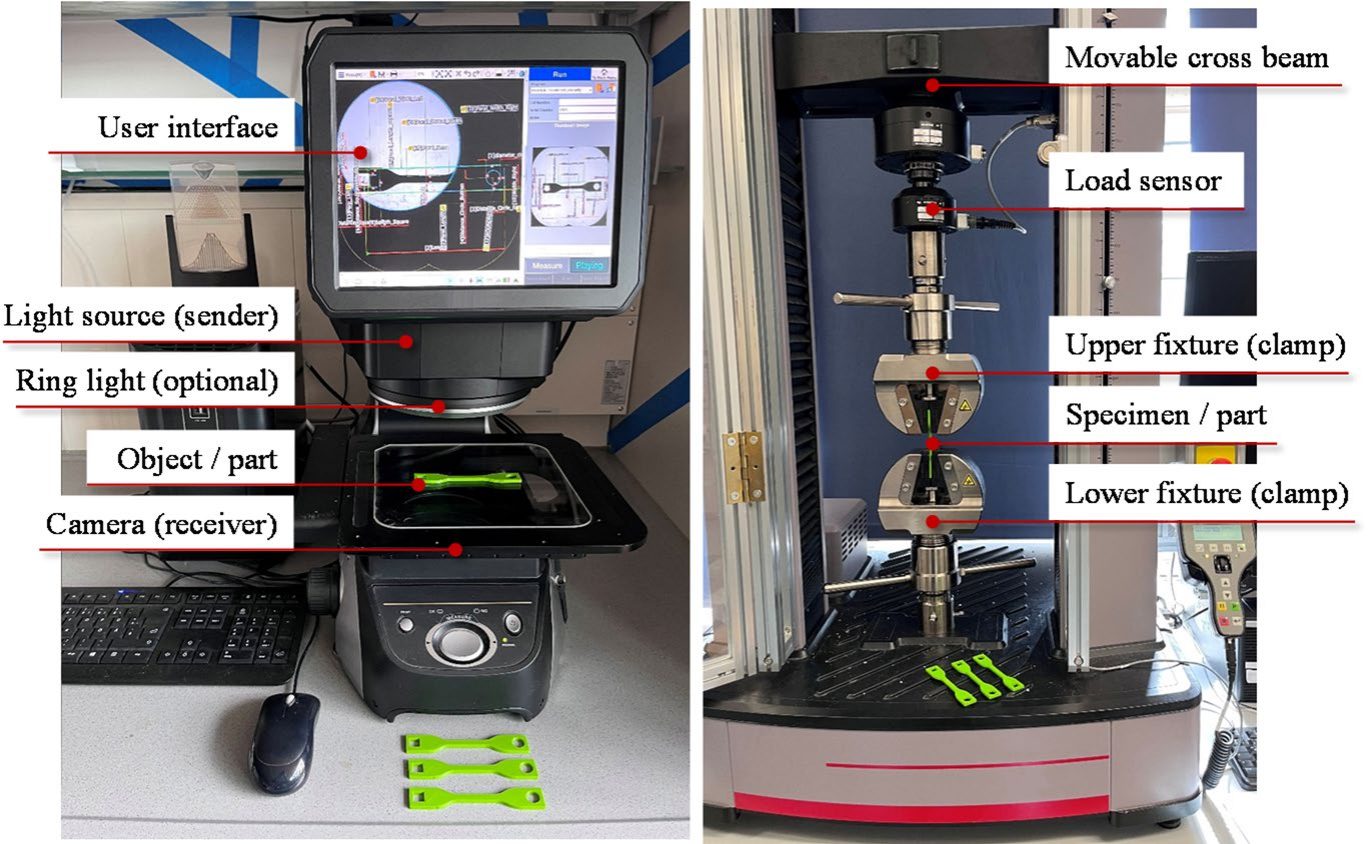
Measurement setup with a part placed in the Image Dimension Measurement System (left) and the tensile test machine (right).

Each printed part was further analysed concerning its dimensional accuracy. Due to the two-dimensional design of the test specimen, an optical measurement technique for 2D applications can be applied. This work used the Image Dimension Measurement System KEYENCE IM‑7000 (Fig. 3)^40,41^. The IM-7000 is made up of a light source that directs light toward a high-precision camera. When an object is positioned between the light source and the camera, light can only pass around the opaque parts of the object. This results in an image where the object’s geometry appears dark, while the surrounding areas remain bright. Because the camera offers very high resolution (detecting differences as small as 2 µm), the edges between dark and bright regions can be recognised with exceptional precision. Consequently, the part dimensions can be accurately measured using integrated image processing and feature recognition algorithms^40^. All steps of the measurement procedure were pre-set in a measurement program, applied to measure all parts. The results are detailed in “results of geometrical analyses of parts (Steps 7–8)” section.

### Step 8: analysis of the roughness of the part

With the Waveline W20 device from the manufacturer Vista Instrument SDN BHD^42^, the tensile specimens’ roughness average Ra on the top surface was measured. The top surface is the one facing along the positive Z-direction building platform. The setup included a ball-tip with a radius of 2 μm and the setup of “program 2”, which follows the ISO 16,610–21 standard^43^. The cut-off was set to 0.8 mm, and the traversing length of the probe on the surface was 4.8 mm. The resulting Ra values are detailed in “Results of geometrical analyses of parts (Steps 7–8)” section.

#### Step 9: analysis of the tensile strength of the part

The tensile tests were carried out with an MTS Criterion 43 tensile testing machine (Fig. 3) using a load cell of 5 kN and following the standard ASTM D638-22^26^ for the definition of the gap between the clamps of 70 mm and the testing speed of 5 mm/min, following the classification of the material described in the ASTM D883^44^. The resulting ultimate tensile strength (UTS) and the true strain are presented in “Results of mechanical analysis of parts (Step 9)” section.

## Results

In this section, we will present the results of the analyses carried out during steps 2 to 9. The section is divided into three subsections, focusing on the material analysis of powder and parts (4.1), the dimensional analysis of the parts (4.2) and the mechanical analysis of the parts (4.3).

To properly contextualise the findings, it is important to briefly compare the characteristics of the powder used in this study (Sinterit PA12 Smooth) with those of widely adopted industrial alternatives, such as EOS PA2200. Both materials are based on polyamide 12 and exhibit similar semicrystalline morphology and thermal degradation pathways^45^. EOS PA2200 is optimised for high-power CO_2_ laser systems and is characterised by narrow particle size distribution, high sphericity, and excellent flowability, supporting consistent densification and surface finish across builds^9,46^.

EOS PA2200 also exhibits optimised melt flow behaviour and minimal batch-to-batch variation due to strict material conditioning and closed-loop process control in EOS systems^16^. Typical mechanical properties include tensile strengths up to 48–52 MPa and elongation at break values exceeding 15–20%, under optimal conditions^47^. These features make it a benchmark material for functional prototyping and end-use components.

The Sinterit PA12 powder used in this study is formulated for compact desktop systems with lower-power diode lasers, and while its morphology may differ slightly in terms of particle roundness or distribution, the base chemical composition and sintering response remain consistent under controlled conditions. Moreover, it has been shown that surface sensitisation strategies can enable diode laser systems to achieve comparable sintering behaviour to CO_2_-based setups^21^. These parallels support the broader relevance of the current degradation and reuse analysis, while acknowledging that differences in hardware, thermal environment, and laser-material interaction must be considered when extrapolating across platforms.

### Results of the material analyses of powder and parts (Steps 2–6)

PA12 is a semi-crystalline polymer, and it exhibits two crystal structures, α- and γ-phases, and usually γ acts as its stable phase. XRD patterns of virgin and 5-times used powders are reported in Fig. 3. The virgin and 5-times reused powder patterns show a single γ (0 0 1) peak at 2θ = 21.5° and two weak reflections of the α-phase at 2θ around 20° and 23.5°, respectively, in agreement with other publications^48^.

The XRD pattern of 5-times reused powder is characterised by a slightly more intense γ (0 0 1) peak compared to that of the virgin powder, and, therefore, the crystallinity and, consequently, the dimensions of the crystals are slightly bigger. Moreover, the reused powder’s γ (0 0 1) peak is shifted to lower 2θ angle values. This indicates that, due to ageing, the powders undergo a deformation due to chain segment rearrangement. Furthermore, the peaks of the α-phase in the reused powder tend to decrease in intensity, thus favouring the formation of the more stable γ-phase.

**Fig. 4:**
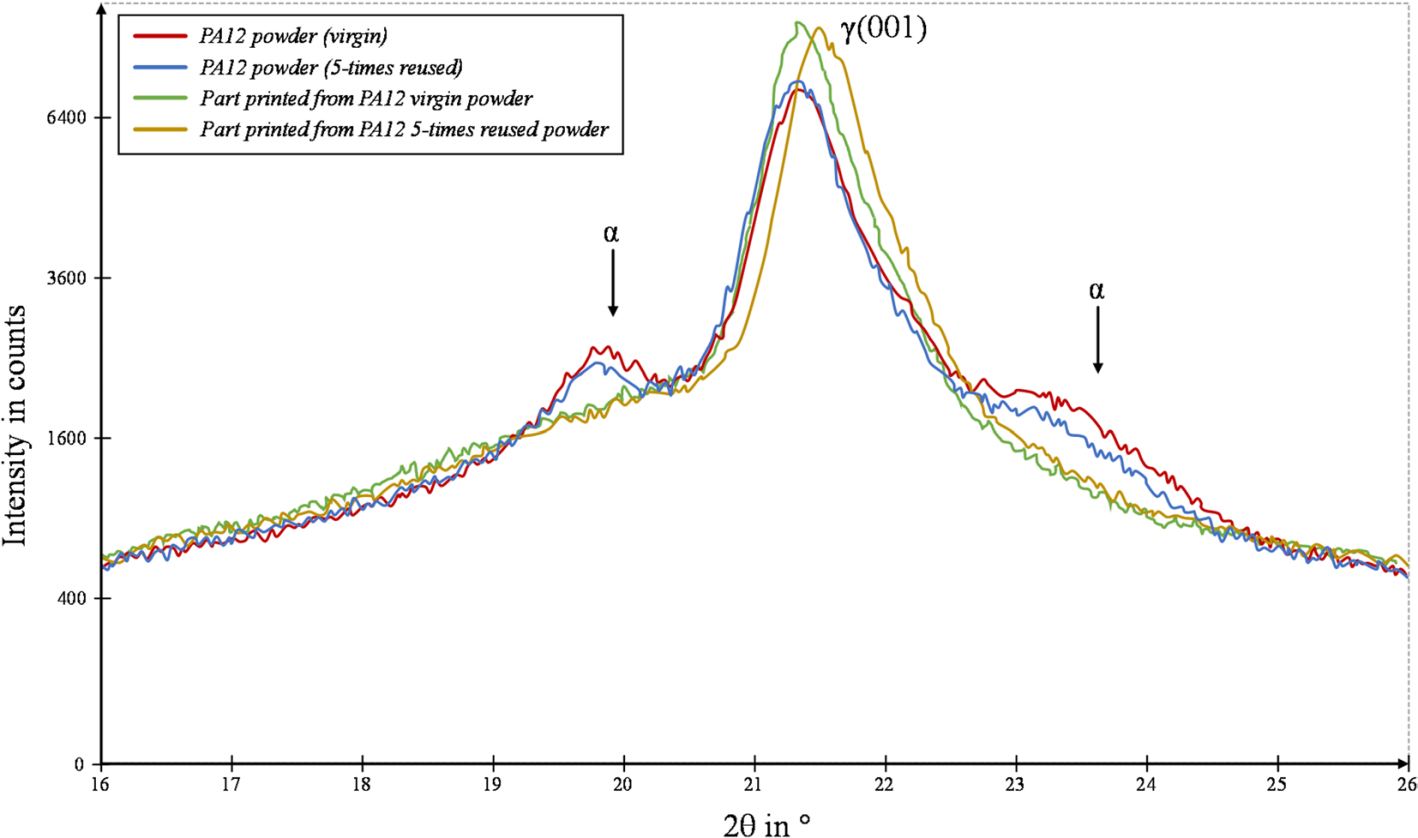
XRD patterns of virgin and 5-times used powder (and of the corresponding printed parts).

The XRD patterns of the parts (taken from the parts printed from both the virgin and 5-times reused powders) are shown in Fig. 4. It details the presence of only the γ (0 0 1) peak, characterized by almost identical intensity and, therefore, a similar crystallinity but greater intensity compared to the corresponding non-sintered powder and therefore by larger crystal dimensions. Moreover, the γ (0 0 1) peak of the parts obtained by sintering the 5-times reused powder presents a shift towards the left compared to the same peak of the sample obtained starting from the virgin powder. This shift indicates an effect of a slight deformation justified by the fact that the sintering of reused powder determines a different rearrangement of the chains compared to that of virgin powder due to a higher molecular weight and viscosity, which disfavour the mobility of the chain^49,50^.

To obtain sintered products of desired quality, it is essential to understand the thermal behaviour of the starting powder since, in the SLS process, the temperature is maintained in the sintering window^47^. The main reason is the adhesion between layers, ensuring good mechanical performances and preventing the powder from melting, avoiding unsintered particles in the parts.

**Fig. 5:**
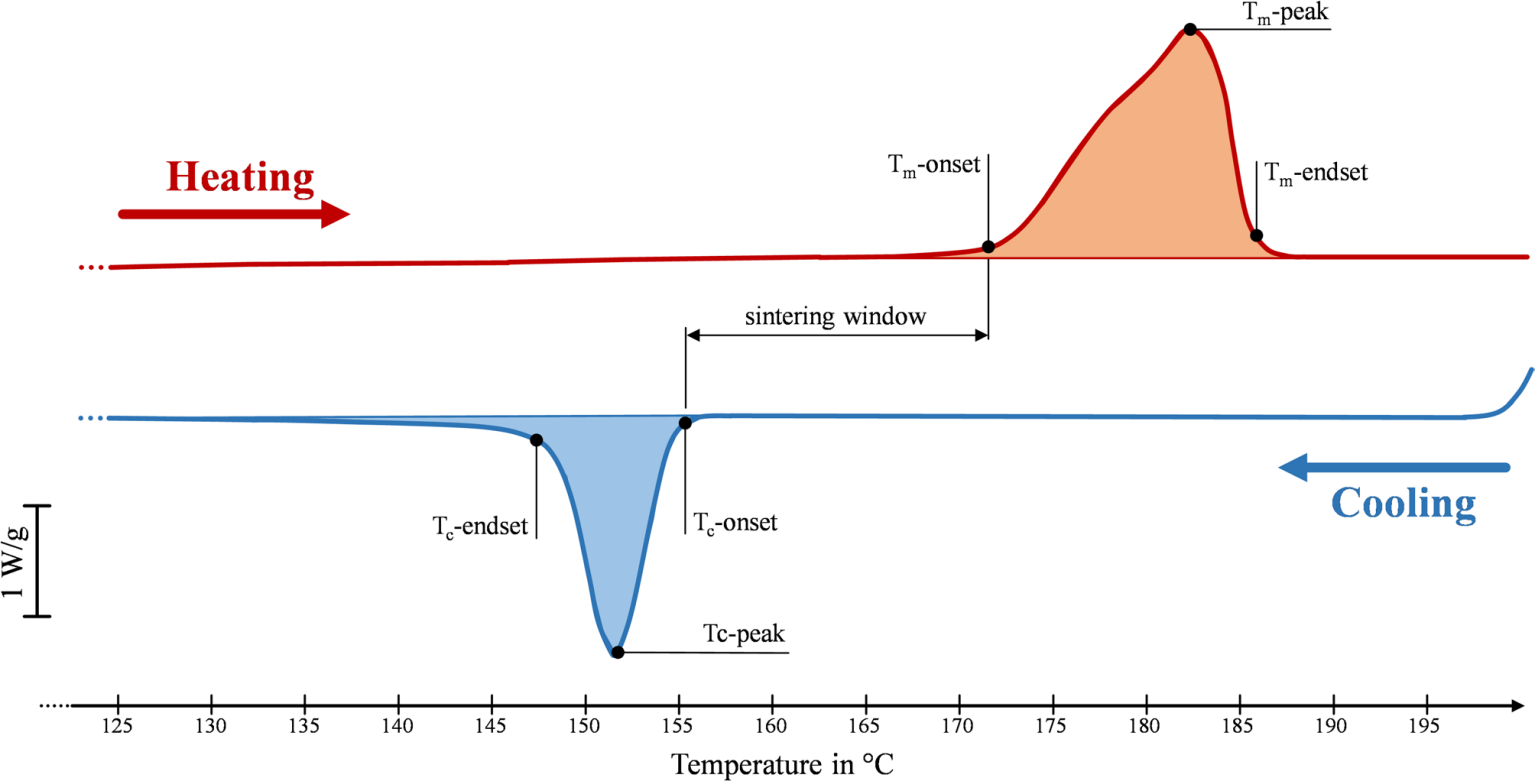
Heating and cooling procedure during the DSC of the PA12 virgin powder.

Figure 5 shows the heating and cooling curves of the virgin powder. The red area represents the energy absorbed during the melting, whereas the blue area represents the energy released from crystallisation. The relevant temperatures related to the melting (index: m) and the crystallisation (index: c), along with energies and the degree of crystallinity from the DSC, are summarised in Table 3.

**Table 3:** DSC results (given values are the mean of the three experiments for each use-cycle).

Indices	Number of reuse cycle of the PA12 powder					
m = melting	0 (virgin)	1-time reused	2-times reused	3-times reused	4-times reused	5-times reused
c = crystallisation						
T_m_-onset (°C)	171.42	175.05	174.68	174.63	174.78	174.39
T_m_-endset (°C)	184.90	185.21	185.41	185.60	185.67	185.98
Melting shoulder (°C)	13.19	9.26	10.72	10.61	10.74	11.09
T_m_-peak (°C)	179.83	180.30	180.75	180.61	180.55	181.02
T_c_-onset (°C)	154.32	154.77	154.88	155.08	155.27	155.49
T_c_-endset (°C)	148.27	148.80	148.83	148.89	148.95	148.84
Crystallisation shoulder (°C)	6.05	6.04	6.05	6.19	6.40	6.65
T_c_-peak (°C)	152.14	152.68	152.71	152.86	152.89	152.93
ΔH_m_ (J/g)	101.71	98.77	98.06	97.37	96.02	94.03
ΔH_c_ (J/g)	58.34	57.54	57.54	57.38	58.00	56.93
Degree of crystallinity (%)	49.05	47.19	46.63	46.39	45.88	44.92

From the virgin powder to the 5-times reused powder, the melting peak T_m_-peak shows an increase of 1.19 °C, while the crystallisation peak T_c_-peak also shows an increase of 0.796 °C. To properly examine the powder deterioration for recycling purposes, these temperature variations do not appear to be sensitive enough^17^. Both melting and crystallisation onset- and endset-temperatures shift from the corresponding temperatures of virgin powder. Even if the shift is a few Celsius degrees, the sintering windows are broad because the change in the particle size makes it difficult to melt them. Moreover, since the mobility of the chain decreases, the enthalpy associated with the melting process ΔH_m_ decreases^51^. The crystallisation enthalpy reduces the energy necessary for crystal development, or ΔH_c_, similarly to how it reduces the melting enthalpy. Degradation activities, including further polymerisation and cross-linking, may be responsible for this decline^19^. At the fifth reuse, the degree of crystallinity of succeeding powders indicates a gradual decrease of 8.42%.

**Fig. 6:**
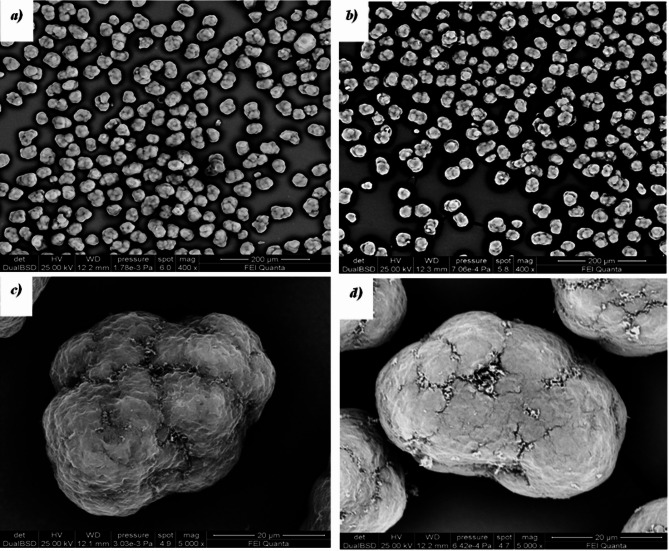
SEM micrographs of virgin powder (**a**, **c**) and 5-times reused powders (**b**, **d**).

Figure 6 shows the SEM micrographs of the virgin powder (*a, b*) and the 5-times reused powder (*b, d*). The particles’ shape appears unchanged and almost spherical/oval (*a, c*). At the same time, the surface morphology of aged PA12 (Fig. 6d) powders is more compact and shows increased cracking and porosity^52,53^ compared to virgin powder (Fig. 6c) due to both the evaporation of moisture and/or alcohol inside the powders when exposed to a long period of pre-heating and sintering processes and to repeated phases of expansions/shrinkages in multiple heating and cooling cycles. Higher porosity in aged powders might also induce some porosity in the produced parts.

**Fig. 7:**
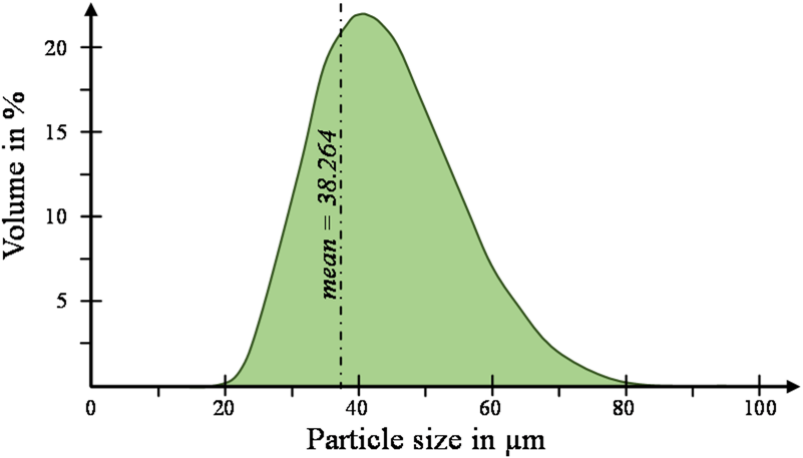
Particle size distribution of virgin powder.

Figure 7 shows the volume distribution of the particle sizes of the virgin powder. The aged powders have nearly identical distributions as the virgin powder, confirming what was observed in the SEM micrographs (Fig. 6a,b). The minimal variation of the mean value of the particle size further justifies this conclusion: 38.264 µm (virgin powder), 38.213 µm (2-times reused powder) and 38.427 (5-times reused powder).

Table 4 shows the density and porosity values of PA12 powders. The porosity increases with the ageing of the powder and undergoes an increase equal to 2.46% for the aged powder, 5 times compared to the virgin one.

**Table 4:** Density and porosity of PA12 powder during different reuse cycles.

	Number of reuse cycle of the PA12 powder		
	0 (virgin)	2-times reused	5-times reused
Bulk density (g/cm^3^)	0.5	0.49	0.48
Bulk density (g/cm^3^)	1.06	1.06	1.06
Porosity (%)	52.85	53.61	54.15

**Fig. 8:**
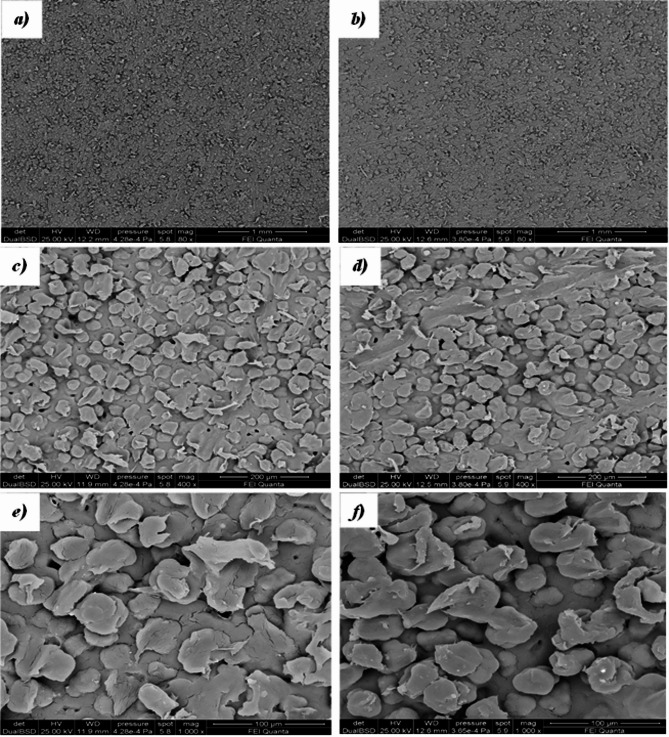
SEM micrographs of top (**a**, **c**, **e**) and bottom (**b**, **d**, **f**) surfaces of the printed part using virgin powder.

**Fig. 9:**
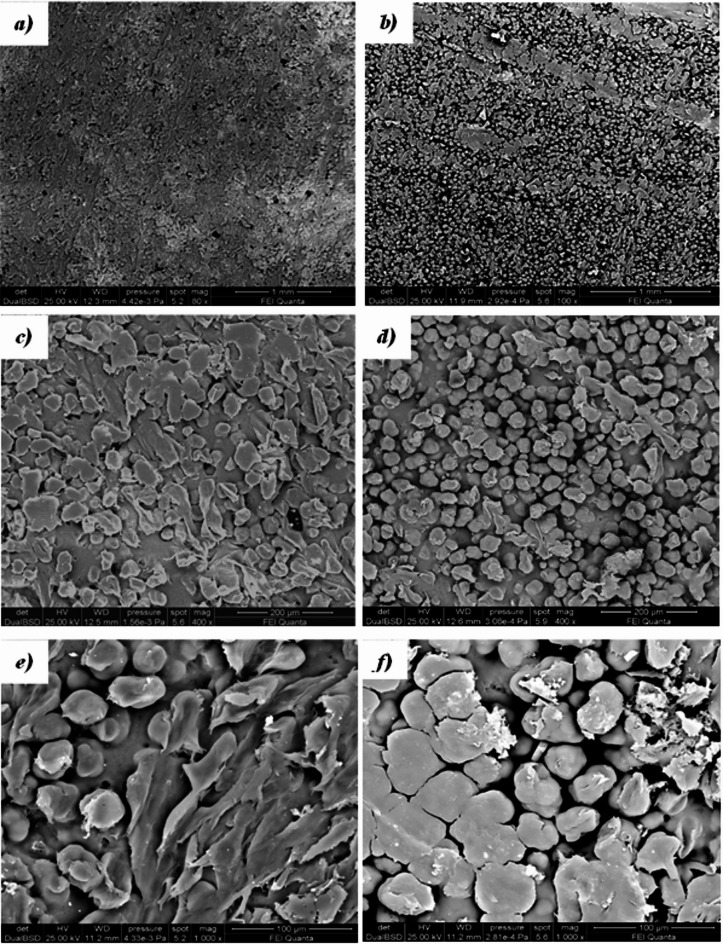
SEM micrographs of bottom (**a**, **c**, **e**) and top (**b**, **d**, **f**) surfaces of the printed part using 5-times reused powder.

Figures 8 and 9 show SEM micrographs of the first sintered layer (bottom surface) and the last sintered layer (top surface) of sintered parts obtained from virgin and the 5-times reused PA12 powders. The top (Fig. 8a,b,c) and bottom (Fig. 8d,e,f) surface morphologies of parts obtained using virgin powder are almost similar, for both surfaces, the PA12 particles and sintering necks are evident, as well as the presence of some voids.

SEM micrographs of the top and bottom surfaces of the specimen obtained by sintering the 5-times reused powder (Fig. 9) show different surfaces. The bottom surface, being the first sintered layer (Fig. 9a,c,e), is characterised by the presence of sintered areas and partially sintered particles with the presence of a sintering neck. A few well-sintered areas are on the last sintered top surface (Fig. 9b,d,f), and the presence is partially sintered. Moreover, many non-sintered powder particles can be seen attached to the surface due to partial fusion, which is responsible for high surface roughness.

**Fig. 10:**
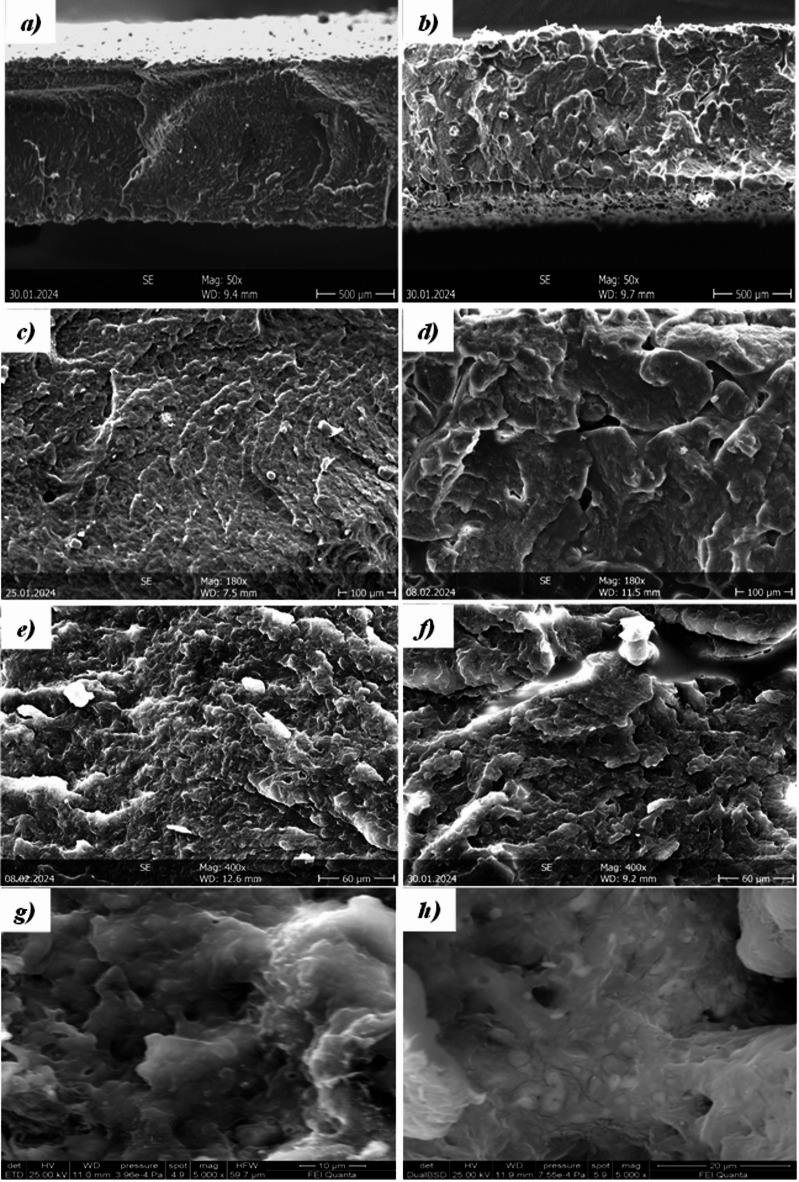
Internal cross-sectional SEM micrographs of the sintered parts obtained using virgin powder (**a**, **c**, **e** and **g**) and 5-times reused powder (**b**, **d**, **f** and **h**).

Figure 10 compares the internal cross-sectional SEM micrographs of the sintered parts obtained using virgin powder (a,c,e,g) and 5-times reused powder (b,d,f,h). The section obtained from the part using virgin powder appears at low magnifications (Fig. 10a), homogeneous and non-sintered particles are detected, unlike the section obtained from the part printed from 5-times reused powder (Fig. 10b). At higher magnifications, the various zones for the section with virgin powder (Fig. 10c,e,g) do not present morphological differences. The surface is spongier than the starting particles and has a lower porosity than the sample’s section obtained with the 5-times reused powder (Fig. 10d,f,h). In the cross-section obtained with the 5-times reused powder, the unmolten particle cores surrounded by spherulites are highlighted (Fig. 10h). These cores are the unmolten central regions of the sintered powders. They occur when the powders do not melt fully due to a lack of energy and appropriate heat absorption.

### Results of geometrical analyses of parts (Steps 7–8)

**Fig. 11:**
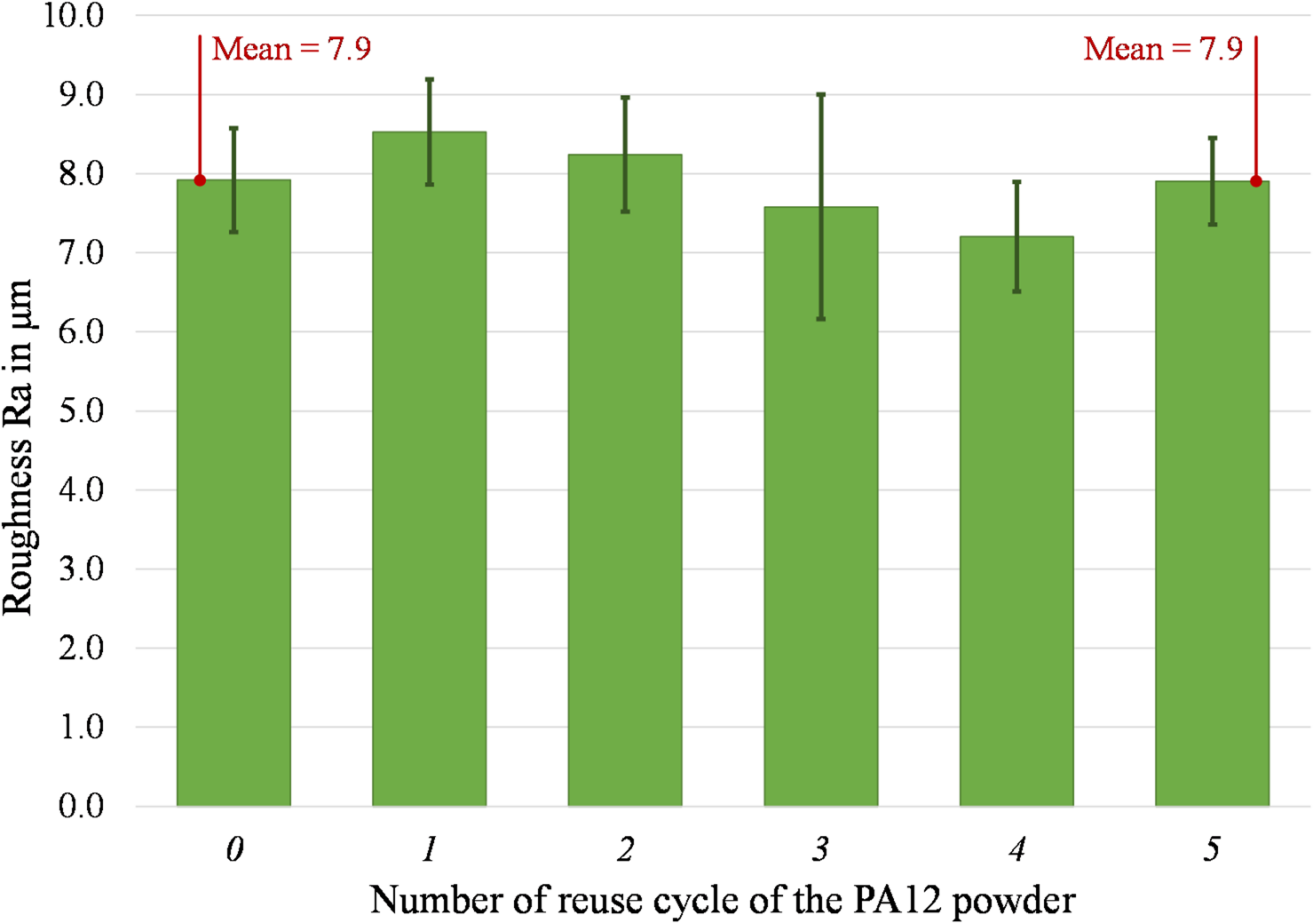
Roughness Ra of the top surface of parts printed from virgin (0) and 1- to 5- times reused powder (1 to 5).

**Fig. 12:**
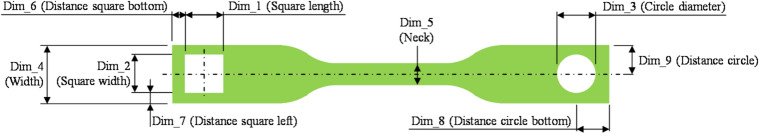
Definition of the relevant dimensions (numbering corresponds to the measurement sequence).

The average roughness of the top surface of the printed parts, along with the standard deviation, is represented in Fig. 11. The average and standard deviation between specimens were considered for every measurement. The results clearly show that the surface roughness underlies no specific trend, as indicated by a less varying mean of Ra. In Fig. 12, the list of relevant dimensions is specified, which are required to evaluate the dimensional accuracy of the printed parts.

**Fig. 13:**
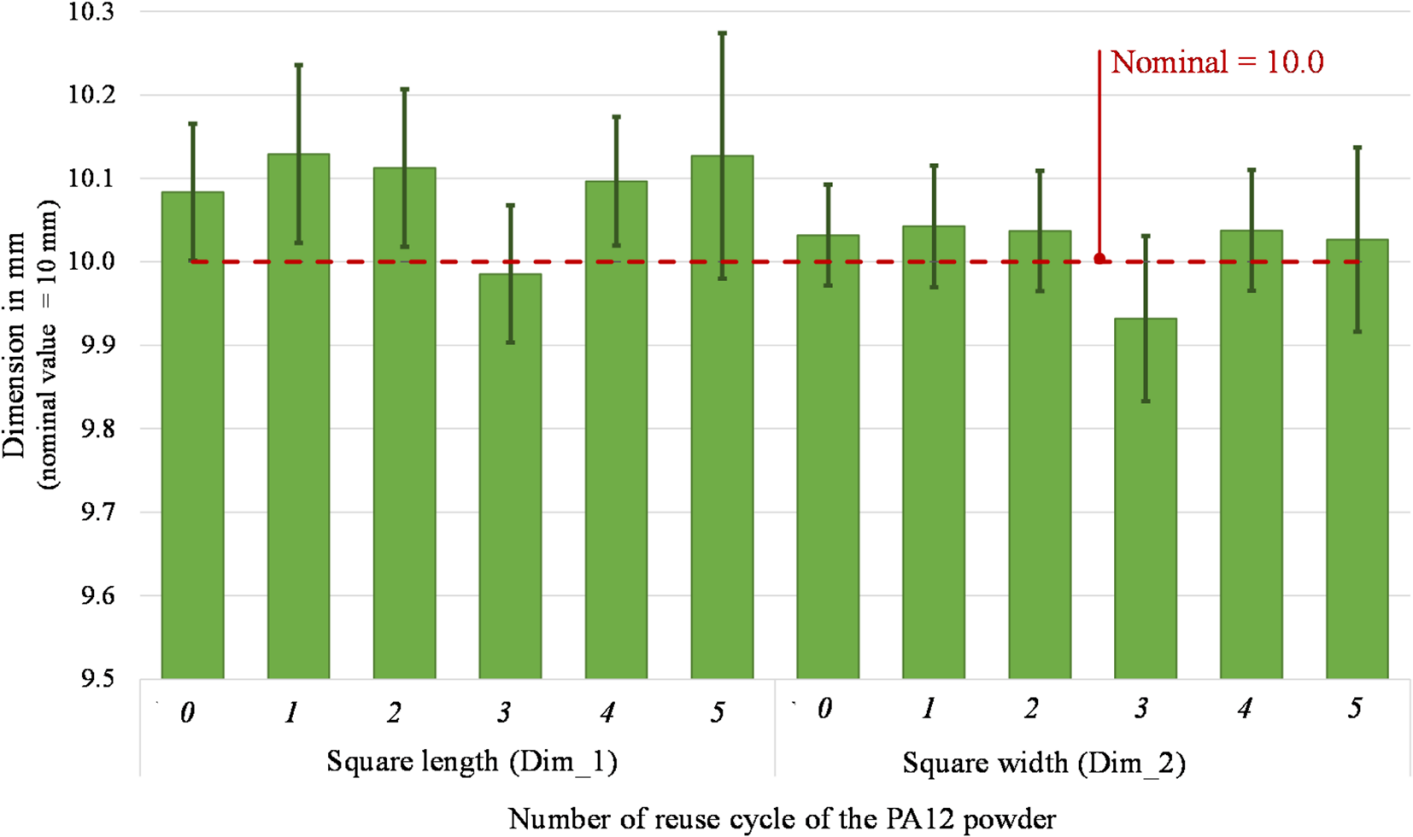
Square length (Dim_1) and square width (Dim_2) of parts printed from virgin (0) and 1- to 5- times reused powder (1 to 5).

**Fig. 14:**
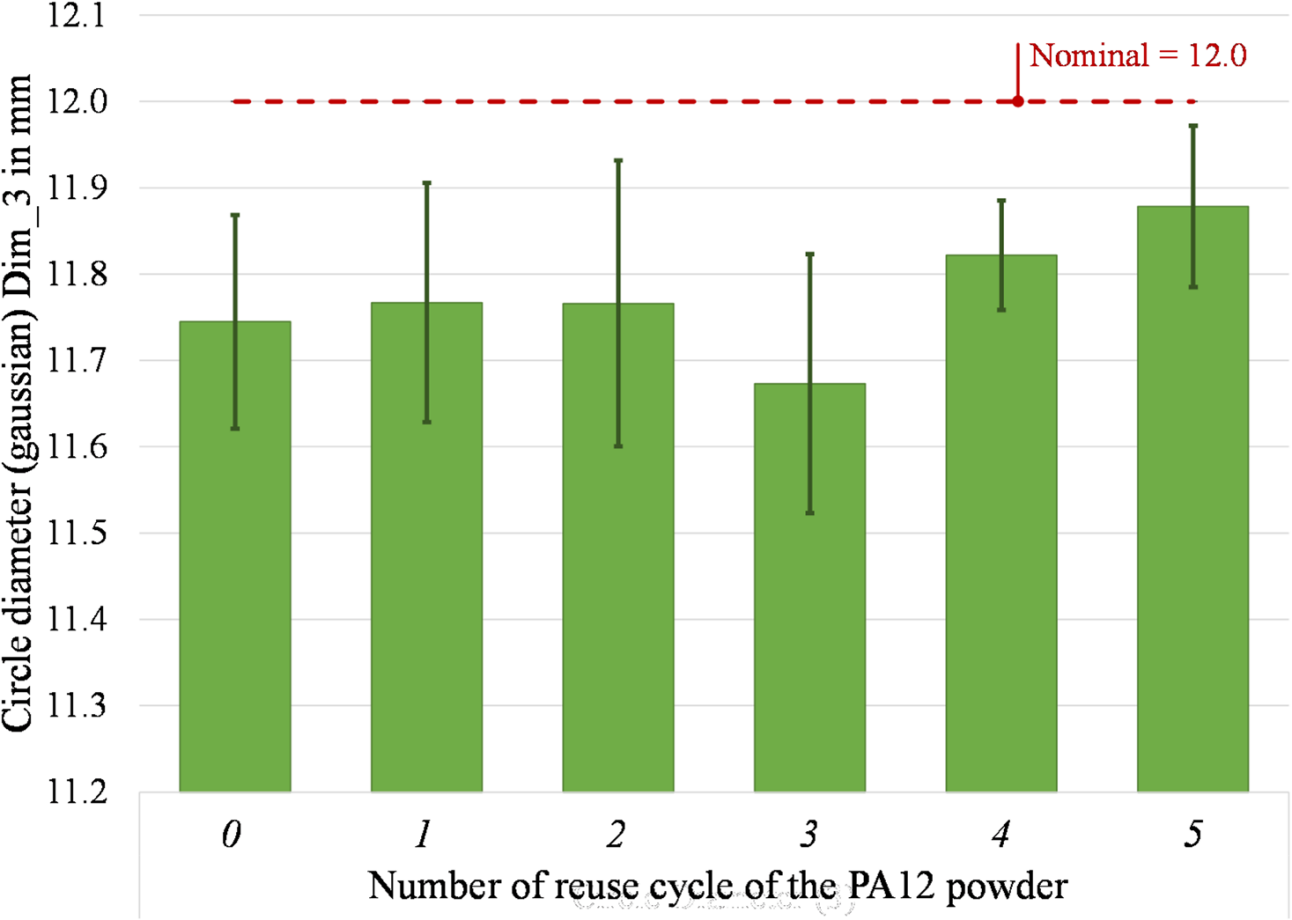
Gaussian circle diameter (Dim_3) of parts printed from virgin (0) and 1- to 5- times reused powder (1 to 5).

The current dimensional analysis was carried out according to the procedure presented in “Step 7: analysis of the dimensions of the part” section and focused on the dimensions of both internal features (square hole and cylindrical hole) and the outer shape of the parts. The length and width of the square hole (Dim_1 and Dim_2) were measured and correlated to its nominal values (Fig. 13). The percentage variation from the nominal square length (Dim_1) ranges between − 0.15 and 1.29% at different reuse stages. The variation of the square width (Dim_2) varies from − 0.68 and 0.42%. Considering the cylindrical hole, its Gaussian diameter (Dim_3) is always smaller than the nominal value, as reported in Fig. 14, varying within the narrow range of − 2.72% and − 1.01%. In all cases, the percentage variations from virgin powder to 5-times reused powder fall inside the estimated error bars.

**Fig. 15:**
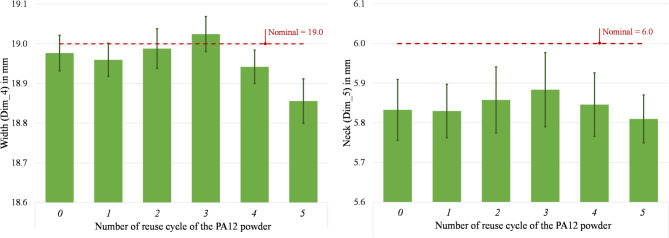
Width (Dim_4) and neck (Dim_5) of parts printed from virgin (0) and 1- to 5- times reused powder (1 to 5).

The dimensions of the width (Dim_4) and the neck (Dim_5) are shown in Fig. 15. The width’s variation from its nominal value is between − 0.76 and 0.13%, while the variation of the neck from its nominal value ranges from − 3.17 to − 1.94%. The percentage values are small, and the ranges of variation are inside the bar errors. Hence, it is possible to declare that for the external features, no significant change occurs due to powder degradation and that the observed shrinkage remains approximately identical during the reuse cycles.

**Fig. 16:**
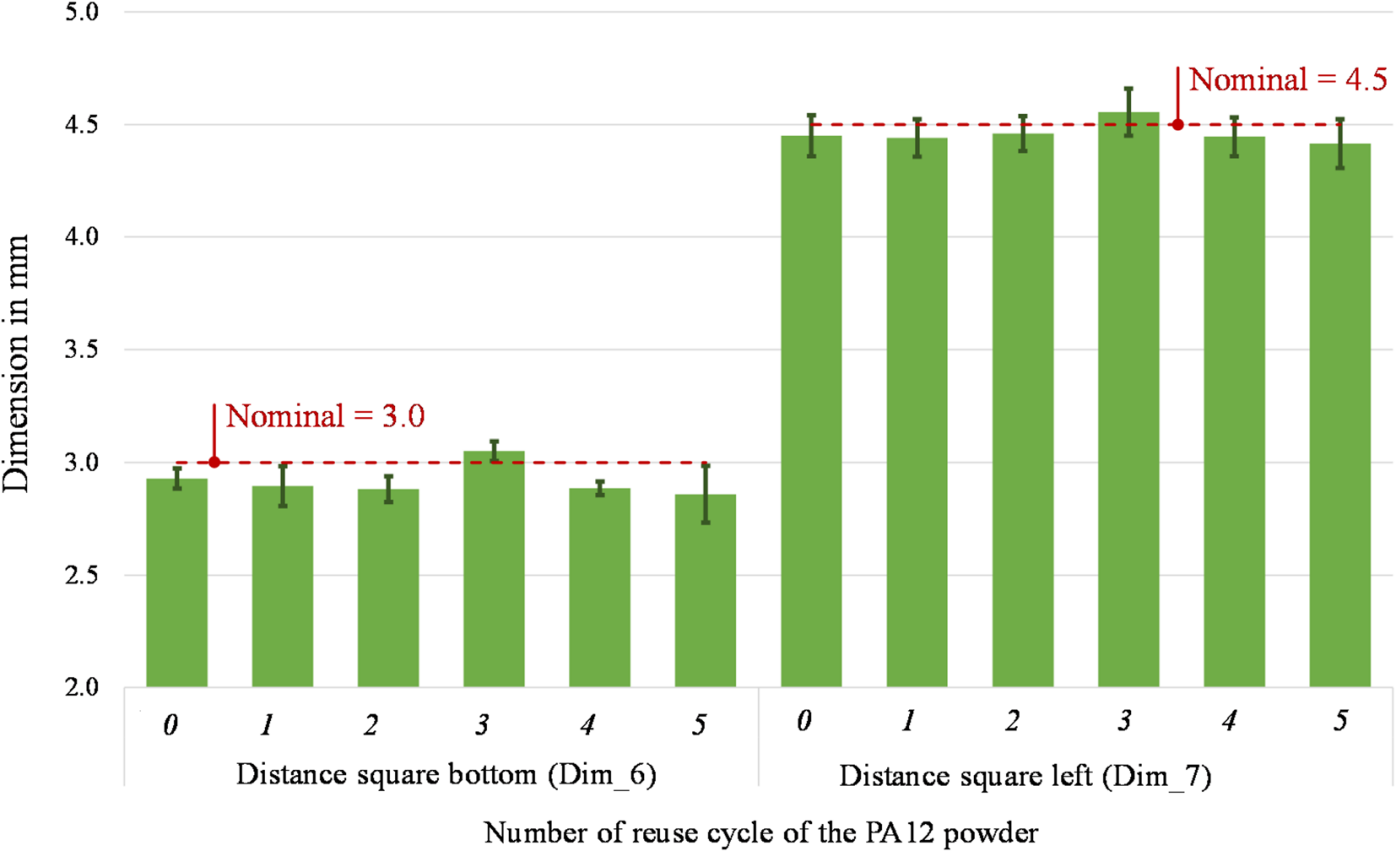
Distance square bottom (Dim_6) and distance square bottom (Dim_7) of parts printed from virgin (0) and 1- to 5- times reused powder (1 to 5).

**Fig. 17:**
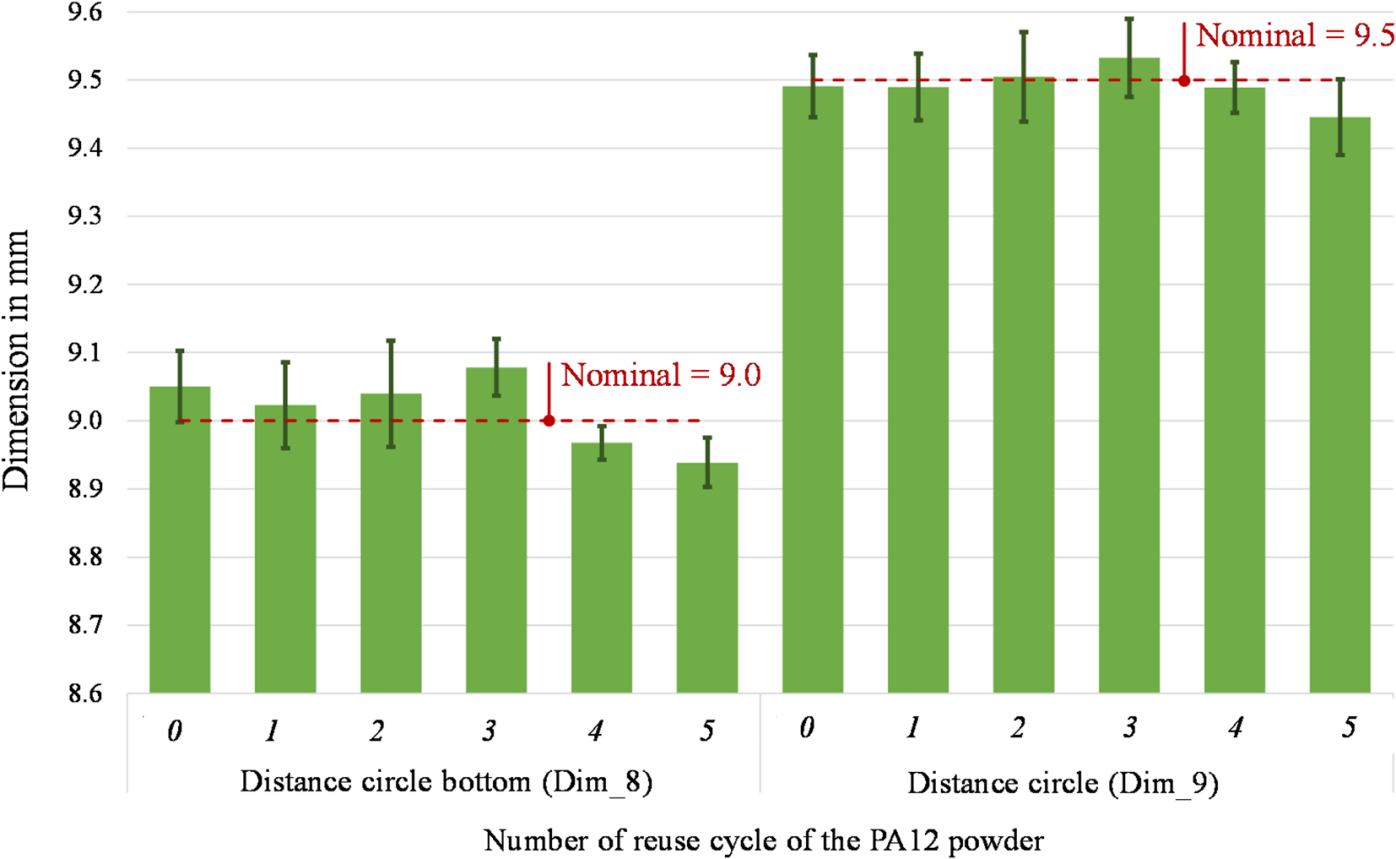
Distance circle bottom (Dim_8) and distance circle (Dim_9) of parts printed from virgin (0) and 1- to 5- times reused powder (1 to 5).

Figures 16 and 17 present the measurements of the four remaining dimensions, which specify the positions of the square hole (Dim_6 and Dim_7) and the cylindrical hole (Dim_6 and Dim_7) in relation to the part’s outer geometry. The percentage variation of the position of the square hole ranges between − 4.69% and 1.71% (Dim_6) and between − 1.89–1.22% (Dim_7). Considering the cylindrical hole, a percentage variation of its centre’s position ranges between − 0.68% and 0.87% (Dim_8) and -0.57–0.34% (Dim_9). These findings correspond to existing research findings in^52^ that show a geometrical variation below 5%. The measuring uncertainty ranges between 0.005 mm and 0.096 mm for the considered dimensions. It coincides with the observed variations of the average values in some cases, thus reinforcing the consideration that there is no significant difference in the dimensional accuracy of the parts printed with either virgin or up to 5-times reused PA12 powder.

An analysis of variance (ANOVA) was performed to evaluate if the multiple reuse of powder may cause the observed dimensional variations^54^. The results (Table 5) proof that a multiple reuse of powder has no statistically significant effect on the dimensional accuracy of printed parts (since all* p*-values are > 0.05).

**Table 5:** ANOVA results on the nine relevant dimensions (DF = Degree of Freedom; Adj SS = Adjusted sums of squares; Adj MS = Adjusted mean squares).

Square length (Dim_1)					
Source	DF	Adj SS	Adj MS	F-value	*p*-value
Powder reuse	5	0.0432	0.0087	0.85	0.543
Error	12	0.1228	0.0102		
Total	17	0.1660			
Square width (Dim_2)					
Powder reuse	5	0.0271	0.0054	0.79	0.580
Error	12	0.0830	0.0069		
Total	17	0.1102			
Circle diameter (Dim_3)					
Powder reuse	5	0.0723	0.0146	0.90	0.512
Error	12	0.1943	0.0162		
Total	17	0.2672			
Width (Dim_4)					
Powder reuse	5	0.0502	0.0101	0.82	0.560
Error	12	0.1472	0.01227		
Total	17	0.1974			
Neck (Dim_5)					
Powder reuse	5	0.0097	0.0019	0.32	0.890
Error	12	0.0725	0.0060		
Total	17	0.0822			
Distance square bottom (Dim_6)					
Powder reuse	5	0.0352	0.0070	0.82	0.557
Error	12	0.1027	0.0086		
Total	17	0.1379			
Distance square left (Dim_7)					
Powder reusing	5	0.0727	0.0145	2.74	0.071
Error	12	0.0637	0.0053		
Total	17	0.1363			
Distance circle bottom (Dim_8)					
Powder reuse	5	0.0120	0.0024	0.88	0.526
Error	12	0.0329	0.0027		
Total	17	0.0449			
Distance circle (Dim_9)					
Powder reuse	5	0.0418	0.0086	3.07	0.052
Error	12	0.0327	0.0027		
Total	17	0.0745			

### Results of mechanical analysis of parts (Step 9)

Anisotropic mechanical characteristics characterise laser-sintered PA 12 parts^55,56^. This study focuses on the tensile characteristics of parts printed perpendicular to the building direction. Three samples were printed for each reuse cycle (0 to 5) and tested in a tensile test machine (Fig. 3). The rupture occurred inside the gauge. The average results of the tensile tests are shown in Table 6, including the mean µ and the corresponding standard deviation σ of the ultimate tensile strength (µ_UTS_ and σ_UTS_), the true strain (µ_Strain_ and σ_Strain_) and the Young’s modulus E (µ_E_ and σ_E_). The (multiple) reuse of powder results in a loss of strength and elongation capabilities. The UTS shows a loss of 35.37% from the use of virgin powder to 5-times reused powder (from 65.3 MPa to 42.2 MPa). Furthermore, the strain is 17.99% lower than it was for the part made from virgin powder. The effect of powder degradation on the Young’s modulus is also significant, resulting in a loss of 30,57% after five reuse cycles of the powder (from 822.66 MPa to 571.18 MPa). These results are in accordance with existing works such as^15,53^. The p-values (< 0.05) of the ANOVA results in Table 7 on the UTS and true strain verify the high correlation of the multiple powder reuse on the mechanical characteristics of the printed parts.

**Table 6:** UTS, True Strain and Young’s modulus of parts printed from virgin (0) and 1- to 5- times reused powder (1 to 5).

Number of reuse cycle of the PA12 powder	UTS		True strain		Young’s modulus	
	µ_UTS_ in MPa	σ_UTS_ in MPa	µ_Strain_ in mm/mm	σ_Strain_ in mm/mm	µ_E_ in MPa	σ_E_ in MPa
0	65.30	0.30	0.25	0.01	822.66	5.17
1	61.97	1.14	0.22	0.03	749.75	13.51
2	50.13	1.02	0.22	0.01	642.13	6.49
3	47.72	0.14	0.20	0.00	627.15	1.77
4	42.11	1.84	0.19	0.01	560.97	17.55
5	42.20	0.00	0.20	0.00	571.18	4.65

**Table 7:** ANOVA results of UTS and true strain of printed parts (DF = Degree of Freedom; Adj SS = Adjusted sums of squares; Adj MS = Adjusted mean squares).

Source	DF	Adj SS	Adj MS	F-value	*p*-value
UTS					
Powder reusing	5	981.929	196.386	202.22	0.000
Error	12	5.827	0.971		
Total	17	987.756			
True strain					
Powder reusing	5	0.0039	0.0008	5.08	0.036
Error	12	0.0009	0.0002		
Total	17	0.0048			

## Discussion

In this section, the results from the previous section are discussed, focusing on the material characteristics of powder and printed parts (in “Material characteristics of powder and printed parts” section), the dimensional accuracy of printed parts (“Dimensional accuracy of printed parts” section) and the mechanical strength of printed parts (“Mechanical strength of printed parts” section).

### Material characteristics of powder and printed parts

The reused powder presents an increase in melting temperature (see Table 3), indicating a preferential crystal form due to crystalline reorganisation. It is in line with other results in literature^6,57^ and can be mainly attributed to the chain-scission degradation mechanism. In fact, the subsequent thermal cycles induce an increase in the amplitude of intramolecular vibrations due to the heat, resulting in their rupture (chain-scission)^58,59^. Less energy can be needed for the molten phase if the powder has partly sintered particle agglomerates. As the powder degrades, some of its temperature history may be retained. Chain scission may also lead to the fragmentation of powder^57^.

According to Table 3, the degree of crystallinity decreases significantly with ongoing powder reuse –from 49.05% (virgin powder) to 44.92% (5-times reused powder). It results from solid-state condensation inside the powder throughout the printing process. The C-O links between the carboxyl and amine groups are broken during post-condensation to create new amide linkages. The effects of chain development and the rise in amorphous content during powder ageing may be to blame for this result^15^. Due to the higher degree of crystallisation in the virgin powder, the higher melting enthalpy suggests that more energy will be needed for the sintering process^60^. Comparable studies using PA12 in CO_2_-laser SLS systems operating under inert nitrogen conditions have shown a more stable crystallinity profile, with decreases below 3% after multiple reuse cycles^17,52^. In contrast, the more pronounced drop observed in this study reflects the influence of open-air processing and lower energy input characteristic of diode-laser platforms, where accelerated thermal oxidation and incomplete fusion contribute to altered crystallisation kinetics.

The spreading of powder plays a critical role in the SLS process. The quality of the powder particle size distribution (Fig. 7) on the bed directly impacts the overall quality of the produced parts. To ensure the deposition of uniform and compact layers of powder, the powder needs to possess good flowability. Reducing the porosity content in the powder with ageing leads to improved mechanical properties. Generally, the layer thickness in the SLS process ranges between 100 µm and 150 μm. Therefore, smooth particles with high sphericity are preferable to achieve the desired microstructure in the final sintered parts ^61^. Meanwhile, the solid density (measured during step 6) is almost stable when reusing the powder. The bulk density presents the same trend of porosity, with a decrease of 2.78%. These results relate to the surface of the particles. With the increment of the reprocessing cycle, the adhesion force between particles increases, and the flowability decreases. Moreover, an uneven distribution of the powder on the bed leads to pores on the printed parts^62,63^.

The different morphologies between the parts obtained by SLS, starting from virgin powder and from the 5-times reused powder, lie in the fact that the unused PA12 powders, during sintering, remain at high temperatures (below the melting point) to a prolonged period. This results in the formation of a denser crystalline morphology and an increase in the melting point of the powder (T_m_-onset, T_m_-endset and T_m_-peak in Table 3) as well as an increase in the chain length due to polycondensation in the solid state, and this justifies their lower sinterability. Furthermore, to confirm what has been said, the sample obtained by sintering the 5-times reused powder is characterised by pores of much larger dimensions compared to those present in the sample sintered using virgin powder (see SEM micrographs in Figs. 9 and 10).

The presence of voids in the part can be directly linked to the decreasing melting energy (Table 3), an index of the missing portion of molten material—starting from 101.71 J/g (virgin powder) to 94.03 J/g (5-times reused powder). Furthermore, the presence of voids leads to weaker parts and thus lower mechanical strength (see tensile test results in Table 6). The mechanical strength of the parts is also linked to the flowability of the powder during the printing process. The chemical structure of each polymer determines its flowability. Polymer chains with simple geometry and short lengths’ slide’ past one another with negligible flow resistance. In contrast, lengthy chains with complicated structures and high molecular weights have higher flow resistance^64^. In fact, works state that a high melt flow rate is due to a lower molecular weight average and molecular number average. In other words, new PA12 powder with shorter chain molecules and higher-order areas might easily create parallel array chains^17^. These trends align with the broader degradation dynamics reported in literature, though systems equipped with high-power CO_2_ lasers operating under optimised thermal conditions often maintain more stable melting energy and produce structurally denser parts even after multiple powder cycles^17,52,57^.

### Dimensional accuracy of printed parts

It is fundamental to reach a good sintering of the powder to obtain a part of sufficient quality. However, with the powder degradation due to its multiple reuses, the melting enthalpy decreases, leading to a decrease in the melting coalescence of the particle and thus, to parts with higher porosity^4,65^. According to literature, the powder degradation leads to various quality problems, such as surface finish issues (orange peel, etc.)^17^ and lower dimensional accuracy. Defining the optimal process temperatures helps obtain parts with no temperature-induced defects, such as shrinkage and curling, since no supports block the part besides the powder itself. Studies on the effect of powder degradation on the part’s geometrical quality found that it decreases by about 5% to 10%^52^. The uncertainty associated with the dimensional deviations in this study was estimated at 58% of their standard deviation. Even though there is a fluctuant variation between positive and negative values of the dimensional deviations from its nominal values, in this study, all observed dimensional deviations are lower than 200 µm. In CO_2_-based systems with inert gas control and optimised scan strategies, the dimensional drift is generally contained within narrower limits, often below 100 µm across several reuse cycles^6,17,52^. Considering the general tolerances according to ISO 2768^66^, the dimensional accuracy of the printed parts (from virgin to 5-times reused power) is still within the best tolerance class “fine” for linear dimensions. In consequence, the effect of the multiple reuses of powder on the dimensional accuracy of printed parts are low and, according to the ANOVA results (Table 5), not significant thus suggesting that compact diode-laser platforms can still ensure acceptable geometric fidelity across multiple reuse cycles, despite operating under more variable thermal and oxidative conditions^26,63^.

### Mechanical strength of printed parts

The weaker layer-to-layer solidification and decreased coalescence of the reused PA12 particles account for the inferior mechanical properties shown in Fig. 10 and^67,68^. The usual crystal structure of PA12 is made up of two phases: α and γ. The more crystalline metastable α-phase is seen in the unprocessed powder. The chain changes into the stable γ-phase during the sintering process. The reduction in crystallinity in components made with old powder is brought about by increased short-molecule production^69^.

Additionally, amorphous areas surround lamellae owing to the material’s low crystallinity. As a result, the matrix’s spherulites are distributed throughout and become coarser. It should be noted that the higher the number of reuse cycles, the rougher-looking spherulites appear due to the aggregation during the growth, which may have increased lamellar thickness. These microstructural alterations will be reflected in the final, significantly lower mechanical strength of the printed parts^62,70^. The decline in mechanical performance observed here is consistent with trends reported for reused PA12 powders. However, studies based on CO_2_ laser sintering systems typically report a slower reduction in tensile strength and elongation at break, especially when processing is performed in nitrogen-controlled chambers^17,52,68^. These systems benefit from deeper melt pools, more uniform energy distribution, and reduced oxidative degradation, which help maintain mechanical properties even after several reuse cycles. In contrast, the open-air diode-laser setup used in this study shows a faster onset of mechanical degradation, in line with reduced sintering efficiency and increased porosity^15,57,67^.

## Conclusion and outlook

This paper aims to investigate the degradation effects of reused PA12 powder in Selective Laser Sintering, focusing on the material characteristics of the powder and printed parts as well as the dimensional accuracy and the mechanical strength of printed parts. An experimental setup was developed to pursue subsequent printing processes, using the unsintered powder from the previous printing cycle as an input. In total, six prints were carried out – starting from the virgin batch of powder to the fifth reuse cycle of the powder.

Initially, the PA12 powder exhibits a well-defined crystalline structure. However, distinctive trends emerge in the X-ray diffraction patterns with each successive printing cycle. The characteristic peak shows a slightly intensified increase and a shift towards lower angles, indicating a crystalline transition from the α to the γ phase. This phenomenon is accompanied by a reduction in the intensity of the α-phase peaks, favouring the formation of the more stable γ phase. Concurrently, the morphological analysis of the particles indicates a structural transformation. Initially, the particles appear nearly spherical or oval, but with further reuse, they become more compact with increasing cracking and porosity. Thermal trends show an increase in melting and crystallisation temperatures. The melting temperature rises by 1.19 °C, while the crystallisation temperature increases by 0.80 °C. These changes indicate a transformation in the thermal behaviours of the powder during printing. Moreover, from the virgin to the fifth cycle of reuse, the degree of crystallinity drops by 8.42%. Successive thermal cycles impact the density and porosity of the powder. Density records a decrease of 2.78%, while porosity exhibits an increase of 2.46%. This may have implications for layer distribution during the printing process. The SEM analysis on parts printed from virgin powder and 5-times reused powder reveals distinct surface morphologies. Ageing was found to cause denser crystalline structures, increased melting points, and longer chain lengths, ultimately reducing sinterability. This leads to challenges such as void formation, larger pores, and weaker parts. The observed effects on the structural and thermal properties of the aged powder involve some worsening in the mechanical performance of the parts manufactured with this kind of powder. In fact, the study`s tensile tests revealed a significant reduction in the UTS by 35.37% and the Young’s modulus by 30,57% after the fifth reuse. Additionally, the strain is notably 17.99% lower than the virgin part, suggesting the potential occurrence of chain scission. Shorter chains exhibit increased mobility, complicating crystallisation and leading to the presence of more amorphous regions, contributing to the observed mechanical deterioration.

Instead, no significant effect may be observed on the dimensional accuracy of the parts. In fact, the nine observed dimensions underlie a low variation due to the reuse of the powder. The maximum deviation from nominal is under 5% and all observed dimensional deviations are lower than 200 µm. Considering the general tolerances according to ISO 2768^66^, the dimensional accuracy of the printed parts (from virgin to 5-times reused power) still fulfils the requirements of the most precise tolerance class “fine” for linear dimensions. As the melting enthalpy decreases, causing reduced particle melting coalescence and producing high porosity components, surface quality changes. Interestingly, the top surface displays a modest variation between the part made with virgin powder and the part subjected to five reuse cycles. Conversely, the bottom part exhibits a more substantial variation, reaching a Ra of 12.64 µm at the fifth reuse compared to the starting value of 6.80 µm in the virgin-made specimens.

This knowledge is critical for implementing an efficient recycling/reuse process of PA12 powder. Future work is needed to improve the efficiency of the powder reuse and to reduce waste while maintaining intact properties. The powder should be subjected to in-depth studies to assess correlations and eliminate thermal history at every stage to ensure the best performance, regardless of the initial state of the powder. Above all, the restoration of mechanical properties needs to be investigated. The adaptation of process parameters to different powder stages could be the subject of future work to see if sintering undergoes improvements in terms of surface and internal, in part, of better cohesion of the particles with a subsequent increase in the degree of crystallinity. Another solution is to investigate material modifications that can improve the stability and resilience of the polymer. This could include adding stabilisers or making alterations to the polymer chemistry.

## Data Availability

The datasets used and/or analysed during the current study available from the corresponding author on reasonable request.
